# Ethical climate and innovative behavior: the mediating role of ethical conduct

**DOI:** 10.3389/fpsyg.2026.1735002

**Published:** 2026-08-05

**Authors:** Bobirjon Alimov, Taekyung Park, Sherzodbek Murodilla Ugli Dadaboyev

**Affiliations:** 1 Bukhara State Medical Institute, Bukhara, Uzbekistan; 2 Department of Business Administration, Yeungam University, Gyeongsan-si, Republic of Korea; 3 Business School, Central Asian University, Tashkent, Uzbekistan

**Keywords:** benevolent climate, ethical behavior, ethical climate, innovative behavior, principled climate

## Abstract

Empirical evidence on the relationship between ethical climate and innovation in public sector organizations remains limited, particularly from a Central Asian perspective. Bridging this gap, this study investigates the influence of ethical climate on innovative behavior, mediated by ethical conduct. Data were collected via surveys from 206 full-time employees. Data were analyzed using regression and Structural Equation Modeling (SEM). Results indicate that a principled ethical climate directly fosters both ethical behavior and innovative behavior. However, a benevolent environment had no significant relationship with ethical behavior. Notably, SEM results revealed that ethical behavior does not mediate the relationship between ethical climate and innovation. These findings suggest that while a principled climate promotes multiple positive organizational outcomes, ethical conduct and innovative behavior act as parallel, independent outcomes rather than a sequential mechanism.

## 1 Introduction

Corporate scandals worldwide have drawn attention to the importance of ethical behavior in organizations (Okpara and Wynn, 2008). Dishonest actions result in billions of dollars in losses, negatively impacting financial markets and consumer confidence (Lewis et al., 2006). Consequently, the relationship between ethical climate and employee attitudes and behaviors has become a popular area of research (Martin and Cullen, 2006).

Ethical climate refers to employees’ shared perceptions of what constitutes ethically correct behavior and how ethical issues should be handled within their organization (Victor and Cullen, 1987). Research suggests a favorable ethical climate can foster desirable organizational outcomes, including organizational commitment, job satisfaction, and innovative behavior (Martin and Cullen, 2006). Since the 1980s, organizations have increasingly started showing interest in ethics and establishing an environment where ethical action would occur. This type of interest was mainly seen in factors such as society’s demand for broader corporate responsibility, regulatory and legal incentives, and financial motivations (Blodgett and Carlson, 1997; Verschoor, 1997). In their study, Morf et al. (1999) found that 75% of the organizations included ethical regulations directly into their mission statements. Moreover, many organizations have not stopped there. Most of them even created specific employees for only ethical purposes and named them as ethical officers.

Victor and Cullen's (1988) Ethical Climate Questionnaire (ECQ) identifies different ethical climate types based on two dimensions: ethical criterion (egoism, benevolence, principle) and locus of analysis (individual, local, cosmopolitan). Benevolent climates emphasize concern for others, while principled climates prioritize adherence to rules and ethical codes (Cullen et al., 2003). Moreover, based on the studies of West and Farr (1989) and Yuan and Woodman (2010), innovative behavior is an individual’s intentional introduction or application of novel ideas, products, processes, and procedures to benefit his or her work role, work unit, organization, or people.

While research has examined the direct effects of ethical climate on various organizational outcomes, fewer studies have explored the mediating role of individual ethical behavior in the relationship between ethical climate and innovative behavior. Therefore, this study aims to investigate whether individual ethical behavior mediates the impact of ethical climate on innovative behavior.

**Figure 1:**
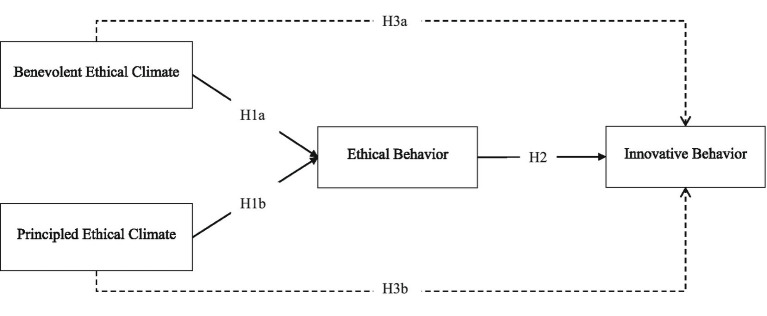
Proposed research model. *H* = hypothesis. Dashed lines indicate indirect effects.

To explain the mechanism through which ethical climate influences innovative behavior, this study anchors its theoretical framework in Social Exchange Theory (SET) (Blau, 1964). SET posits that employment relationships are built on the principle of reciprocity. When employees perceive that their organization provides a supportive or highly principled ethical climate, they feel a reciprocal obligation to respond positively. We argue that this reciprocation first manifests as adherence to ethical behavior. Furthermore, because a strong ethical climate fosters trust and reduces the fear of unfair punishment (Sarwar et al., 2025a), it creates the psychological safety required for employees to engage in extra-role behaviors, such as innovation. While the direct link between climate and innovation has been explored, the mediating role of ethical behavior remains a critical research gap. By unpacking this mechanism, we aim to demonstrate that ethical conduct is a vital stepping stone toward organizational innovation. Figure 1 shows the proposed research model.

## 2 Literature review and hypotheses development

### 2.1 Ethical climate and ethical behavior

Recent literature strongly supports the idea that institutional environments shape employee actions. For instance, institutional support and positive environments are critical drivers of forward-thinking aspirations, such as entrepreneurial and innovative intentions (Sarwar et al., 2025c). Similarly, a robust ethical climate has been proven to significantly foster sustainable workplace behaviors by acting as an intrinsic motivator (Sarwar et al., 2025a). Conversely, when the workplace climate is negative—such as environments characterized by abusive supervision—employees are much more likely to disengage morally and exhibit deviant behaviors like cyber-loafing (Sarwar et al., 2025b). Taken together, these contemporary studies highlight that an organization’s climate dictates the behavioral trajectory of its employees.

Extensive literature establishes that ethical climate significantly influences employee attitudes and behaviors (e.g., Barnett and Vaicys, 2000; Ford and Richardson, 1994; Victor and Cullen, 1988). Ethical climate refers to an employee’s general perception of an organization’s operations and procedures to promote ethical behavior (Victor and Cullen, 1988). Ethical climate represents the organizational values, practices, and procedures that pertain to moral behaviors and attitudes (Victor and Cullen, 1987). Because most theoreticians see attitudes related to moral issues as the most compelling for driving behavior (Kohlberg, 1967), one can expect that the individual’s perception of ethical, morally relevant organizational values, practices, and procedures should substantially affect people’s affective reactions to the organization. An ethical climate can improve relationships within an organization, employees’ attitudes and behaviors, and enhance the performance of organizations (Elci and Alpkan, 2009). Employees who perceive an ethical climate in organizational policy will likely become involved in activities that help organizations boost their performance.

The basis for a relationship between ethical climate and behavior stems from the ethical decision-making criteria that underlie the empirically derived dimensions of ethical climate. If the ethical climate dimensions promote decision-making based on a high regard for all persons affected by organizational decisions, policies, and practices, then one would expect the behavior of employees to be more ethical (Victor and Cullen, 1988). Thus, ethical behavior would be expected to be promoted only by benevolent and principled climates based on deontology and utilitarianism principles. More unethical behavior would be expected to exist in organizations or organizational subunits where decision-making is based on egoistic principles, the foundational principle for the egoistic dimension of ethical climate.

This study specifically focuses on positive ethical behavior rather than unethical deviance, thereby examining the moral climate of organizations from the perspective of local benevolent and principled climates. An egoistic climate is not considered a part of our focus here.

The positive impact of a benevolent ethical climate on ethical behavior is consistent with the ethical theory of utilitarianism (Barnett and Schubert, 2002). In this sense, utilitarianism aims to maximize the utility or happiness of the greatest number of people (Bentham, 1815). Additionally, utilitarianism is more about consequences in nature as it focuses on the results of behavior and not on the character of the actor or the action (deontological ethics). Consistent with the utilitarian theory, a benevolent climate offers a caring environment. When all organization members care for each other, they are more likely to behave ethically, as they see the other members’ values as similar to theirs. In other words, the organization’s members enjoy working in this environment. This enjoyment may lead to acting ethically. As a result, everyone will be expected to behave within ethical standards (Giacalone and Jurkiewicz, 2015).

Furthermore, a benevolent climate requires employees to carefully consider the consequences of their actions and decisions on the referent group, both within and outside their organization. In a benevolent environment, every member of the organization is concerned with the well-being of other members (Parboteeah and Kapp, 2008). Moreover, though employees may not consider their actions morally wrong or serious, the benevolent climate encourages them to evaluate them within a broader scope of how they could impact others (Barnett and Vaicys, 2000).

Principled climate is one of the two climates we embraced in this study. Unlike the previous two climate types, this climate puts emphasis not on the happiness or pleasure of the individual or group, but rather on abstract impersonal rules of conduct (Kant, 1993; Kohlberg, 1969). These rules demonstrate themselves in the form of personal morality, organizational regulations and procedures, and legal and professional codes of conduct. Most researchers find this dimension similar to the integrity dimension of trust, which refers to the trustor’s perception that the trustee adheres to a set of principles related to fairness and integrity deemed acceptable to the trustor (Mayer et al., 1995).

Victor and Cullen's (1988) ethical criterion of principle embodies the application or interpretation of rules, laws, and standards in the normative expectations of a social unit. When faced with a moral dilemma, organizational or group norms suggest that the decision-maker resort to decisions based on adherence to rules and codes (Cullen et al., 2003). The expected sources of principles for such moral reasoning can be internal to an individual with a principled-individual climate or external, such as with a local ethical or broader code (Victor and Cullen, 1988). When organizations develop principled climates, they are more likely to be consistent with internalized professional norms and values, leading to greater organizational commitment. Suppose the individuals show a higher level of commitment. In that case, they are more likely to devote themselves to their organizations, act within their ethical standards and rules, and do their best to benefit them. Accordingly, we hypothesized the following:

*Hypothesis 1a*: A benevolent climate is positively related to ethical behavior.

*Hypothesis 1b*: A principled climate is positively related to ethical behavior.

### 2.2 Ethical behavior and innovative behavior

While unethical behavior brings harm, violates principles, and does damage, ethical behavior usually refers to rule following and an absence of harm, behaving consistently, being trustworthy, not damaging others, and supporting or investing in the system (Ferrell and Gresham, 1985; Hunt and Vitell, 1986). However, it is not all about ethical behavior. If ethical behavior crosses the border of avoiding harm and rises to the next level, it may become virtuous. Terms such as honor, goodness, and ennoblement describe the condition of virtuousness (Caza et al., 2004).

It is important to draw a conceptual distinction between ethical behavior and virtuous behavior. Ethical behavior generally refers to adherence to established normative standards, rule-following, and avoiding harm to others or the system (Ferrell and Gresham, 1985). It serves as the baseline of acceptable organizational conduct. In contrast, virtuous behavior crosses the boundary of simply ‘doing no harm’ and moves into the realm of proactive goodness and ennoblement (Caza et al., 2004). In the context of this study, standard ethical behavior acts as a foundation. When employees act ethically, they establish a secure, predictable environment. This psychological security is a prerequisite for innovation, as innovative behavior inherently involves risk-taking, challenging the status quo, and vulnerability (Amabile et al., 1996).

Grounded in Caza et al. (2004), we refer to virtuousness here as the behavior that is the next level of ethical behavior and a behavior that benefits the organizations and people. Individuals aspire to be virtuous at their best (Caza et al., 2004). Lipman-Blumen and Leavitt (1999) stated that conditions of virtuousness represent conditions of flourishing and vitality. It is the basis of moral values, willpower, and stamina in the face of challenge (Baumeister and Exline, 1999; Emmons, 1999). It supports ethics as a stable point by being the underlying standard of goodness that motivates the ethical rule.

The literature shows a positive link between positive feelings, emotions, and mood and employees’ innovative behavior. As we mentioned, employees in organizations with an ethical climate behave within ethical standards and try to stick to their organization’s ethical rules and principles. As a result, these employees may feel proud of their organization. Thomas et al. (2004) argue that employees working for ethical organizations have strong pride in their organization, because they consider that they have a competitive advantage over other organizations. Moreover, that means employees may be in a positive mood when they belong to an ethical organization and act ethically, showing a positive approach towards its ethical rules.

According to Choi and others (2013), positive moods facilitate creative problem-solving, which was argued by several studies (e.g., George and Zhou, 2007; Isen et al., 1987). Isen et al. (1987) showed that positive moods enhanced employees’ creative problem-solving ability. Experiencing ethical behavior and virtuous behavior in organizations produces positive emotions, enhancing innovativeness (Cameron et al., 2004). People increase their interest in and accessibility to new ideas and information because of being in a state of inspiration, gratitude, and other positive emotions. They become more creative (Isen et al., 1987). Thus, we hypothesize the following.

*Hypothesis 2*: Ethical behavior is positively related to innovative behavior.

### 2.3 Ethical behavior as a mediator

Ethics is becoming an excellent tool for organizations and firms to strive and survive in the competitive world, and it protects them from unwanted catastrophes (Beauchamp et al., 2004; Ferrell et al., 2008). It helps organizations stay on track to accomplish their vision and mission. The priority for all organizations (whether private or public) is to bring “added value.”

By creating an appropriate environment for the employees, organizations may urge them to act ethically. However, acting ethically should not be the last point or stop. Because ethical behavior is the opposite of unethical behavior, it means not involving oneself in unethical behavior (Ferrell and Gresham, 1985). Consequently, the ethical behavior of employees should turn into virtuous behavior (that is, an ethical behavior with positive effects) that finally benefits the organization they are working for.

Scott and Bruce (1994) examined how contextual and individual difference variables impact innovative behavior through the perceptions of organizational climate for innovation, which was conceptualized as affecting innovative behavior because it signals expectations and potential outcomes of behavior. Here, we add ethical behavior with a positive effect (virtuousness) that consequently causes innovative behavior. To cope effectively and perform successfully in turbulent conditions, individuals and organizations must avoid harming – that is, they should stick to ethical rules and act virtuously, fostering the best of the human condition (Caza et al., 2004).

We argue that two types of ethical criteria, dimensions of ethical climate (benevolent and principled), positively impact innovative behavior through ethical behavior. Saini and Martin (2009) used benevolent and egoistic climates to examine the relationships between ethical climate and employees’ risk-taking propensity. As risk-taking is valued and “treated as essential to innovation” (March and Shapira, 1987, p. 1413) and innovation is impossible without taking a necessary amount of risk, its role is crucial in forming innovative behavior. Saini and Martin (2009) found that an egoistic ethical climate was negatively related to risk-taking propensity, and a benevolent ethical environment was positively related to risk-taking propensity. To explain their finding, they suggested the possibility that employees in organizations with a benevolent ethical climate would more safely and confidently take risks, because they had fewer concerns about penalties for failure from the organization. This means that employees perceiving their organization as having a benevolent ethical climate, characterized by concern for others’ interests or well-being, are first eager to behave ethically and then take risks more confidently, as long as they follow ethical standards. Given that employees’ risk-taking activities are an essential factor for innovation and innovative behavior (Amabile et al., 1996; Mumford, 2000), it is expected that organizations with a benevolent ethical climate will demonstrate more risk-taking behavior for innovation.

When faced with an ethical dilemma in the principled climate, individuals pursue principles grounded in adherence to rules, procedures, and codes (Cullen et al., 2003). Organizations make rules, standards, and procedures based on their culture and climate. Here, individuals make decisions that align with their organization’s standard rules and guidelines (at the local level). Sticking to the company rules and procedures is a sign of acting ethically. By doing this, employees devote themselves to their organizations and do their best to benefit them by engaging in extra-role activities, such as innovative behavior. In other words, sticking to standard rules and procedures facilitates employees to engage in innovative behavior. Thus, we set another two hypotheses as follows:

*Hypothesis 3a*: Ethical behavior mediates the relationship between benevolent climate and innovative behavior

*Hypothesis 3b*: Ethical behavior mediates the relationship between principled climate and innovative behavior

## 3 Methodology

### 3.1 Data collection

Data were collected from the employees employed at public sector organizations, such as state-stock joint companies, joint-stock commercial banks, various ministries, research centers, and institutes in Tashkent city, Republic of Uzbekistan. A questionnaire survey was administered to the employees of these organizations. Anonymity of responses was promised. The questionnaire was written in the two languages, English and Uzbek, since Uzbek is a commonly used language in Uzbekistan. We used Brislin’s (1980) method, which involved translating the scales from English into Uzbek as mentioned earlier. First, the items were translated from English into Uzbek by two persons who are fluent in both languages. Second, one of them conducted blind back-translation, and then we compared the first and the second versions, checked for mistakes, found out parts of concern, and modified the questions.

Out of a total of 250 distributed questionnaires, 224 questionnaires were returned. Eighteen questionnaires were excluded due to incomplete or missing data, bringing about a usable sample with 206 respondents, yielding % response rate of 82.4%. Since this study employed a self-report questionnaire to collect data, findings might be biased due to measuring multiple constructs using the same respondent source, and lead to the standard method variance (CMV) problem. This study employed the recommended approaches to minimize the potential CMV (Podsakoff and Organ, 1986; Podsakoff et al., 2003). Respondents were assured of anonymity and the confidentiality of their responses and told that there were no right or wrong answers, which encouraged them to answer as honestly as possible.

Table 1 shows the characteristics of the respondents in the sample. The vast majority of the respondents were in the age group between 18 and 49 years old (185, or 90%), 149 (72.3%) were male, and 57 (27.7%) were female. Most respondents were staff members (136, or 66%). Regarding working experience, 99 (48%) had at least 5 years. Regarding educational background, 190 (91%) had an undergraduate degree.

**Table 1:** Sample data characteristics.

Respondents	*N*	%
Gender		
Male	149	72.3
Female	57	27.7
Age		
18–29	70	34
30–39	85	41.3
40–49	30	14.6
50–59	16	7.8
60 or more	5	2.4
Marital status		
Married	151	73.3
Not married	55	26.7
Educational background		
Technical college/lyceum	16	7.8
Undergraduate	90	43.1
Graduate	98	47.6
Other	2	1.0
Work experience (tenure)		
Less than 1 year	27	13.1
1–4 years	80	38.8
5–9 years	49	23.8
10 years or more	50	24.3
Rank		
Staff	136	66.0
Deputy head of department	19	9.2
Head of department	17	8.3
Director	4	1.9
Other	30	14.6

*N* = 206.

### 3.2 Measurements

#### 3.2.1 Ethical climate

The instrument used for this research was developed by Victor and Cullen (1987, 1988, 1990) and has an 8-item Ethical Climate Questionnaire. The measure classifies two climate types (benevolent local and principled-local), each measured using a five-point Likert scale, where one strongly disagrees and five strongly agree. Thus, a low score represents the total absence of a climate, and a high score represents the total presence of a climate. We asked the participants to answer questions regarding the climate of their organization and not how they would want the climate to be (see Table 2).

**Table 2:** Validity and reliability metrics.

Variable	Items	Cronbach’s ***α***	Composite reliability (CR)	Average variance extracted (AVE)
Benevolent climate	3	0.66	0.772	0.532
Principled climate	4	0.71	0.775	0.466
Ethical behavior	5	0.62	0.749	0.435
Innovative behavior	6	0.87	0.890	0.575

#### 3.2.2 Ethical behavior

In measuring ethical behavior, we try to measure the degree to which a person is likely to behave in a particular or unethical manner. The assumption is that the people responding to a stimulus, or a question, know whether the behavior they indicate is ethical or unethical. Most researchers find it impractical to observe unethical behavior for research purposes, and most usually use one of two methods (Oz, 2001). The first method is to describe a scenario of unethical behavior and ask the respondents to indicate to what degree they agree with the behavior or to provide a scenario with an ethical dilemma and ask the respondents to indicate how they would behave, with closed-ended potential answers which are pre-ranked by degree of ethicality (Swinyard et al., 1990). The second method is to provide first-person statements of (un)ethical behavior and ask the respondents to indicate to what degree they agree or disagree with the statements (Oz, 1990; Akaah, 1993).

This research used the second method to provide the respondents with first-person statements. The ethical behavior instrument was adopted from Fu and Deshpande (2012). Here, the ethical behavior was measured based on respondents’ perceptions of their moral behavior. Some modifications were applied to the scale previously used by Fu and Deshpande (2012). The statements of Fu and Deshpande were directed to the second person, and we changed them to the first-person statement by putting “me” instead of “you.” Additionally, we changed some statements from positive to negative to avoid reverse coding issues. Some statements are as follows: “It is not acceptable for me to make personal calls at work”; “It is not acceptable for me to take office supplies home”; “To get ahead in my future career, I should not compromise my ethical standards.”

#### 3.2.3 Innovative behavior

The instrument of innovative behavior used for this research was developed by Bysted and Hansen (2015). Some statements are as follows: “I am creating new ideas for improvements”; “I am often searching out new working methods, techniques, or instruments”; “My ideas generate original solutions to problems.” All items asked the participants to indicate their answers on a five-point Likert-type scale (1 strongly disagree and five strongly agree), the degree to which they agreed with statements.

#### 3.2.4 Control variables

Based on previous research (Jung et al., 2003; Mumford et al., 2002; Yidong and Xinxin, 2013; Bysted and Hansen, 2015), we controlled for several variables to reduce the potential impact of unmeasured variables on innovative behavior. We controlled for demographics, which are age and gender. Work tenure was also used as a control variable.

### 3.3 Measurement model, reliability, and validity

To evaluate the construct validity and overall fit of our measurement model, a Confirmatory Factor Analysis (CFA) using maximum likelihood estimation was conducted. The results indicated that the hypothesized four-factor measurement model fit the data excellently: $\chi^{2}$ (128) = 172.34, *p* = 0.005, $\chi^{2}/\mathrm{df}$ = 1.35, Comparative Fit Index (CFI) = 0.96, Tucker-Lewis Index (TLI) = 0.95, Root Mean Square Error of Approximation (RMSEA) = 0.04 (90% CI [0.02, 0.06]), and Standardized Root Mean Square Residual (SRMR) = 0.05. All indices successfully met or exceeded the recommended thresholds for acceptable model fit (e.g., CFI and TLI ≥ 0.90, RMSEA ≤ 0.08, SRMR ≤ 0.08; Hu and Bentler, 1999), demonstrating that the study variables are empirically distinct.

To assess internal consistency, we evaluated Cronbach’s alpha ( α ) alongside Composite Reliability (CR). Cronbach’s α for innovative behavior (0.87) and principled climate (0.71) exceeded the traditional 0.70 threshold. While benevolent climate (0.66) and ethical behavior (0.62) fell slightly below 0.70, values above 0.60 are considered acceptable in exploratory research or for scales with fewer items (Hair et al., 2010; Nunnally, 1978). More importantly, as SEM relies more heavily on indicator loadings, the CR values for all constructs (ranging from 0.749 to 0.890) comfortably exceeded the recommended 0.70 benchmark, confirming robust internal reliability. Furthermore, discriminant validity was evaluated using the Heterotrait-Monotrait (HTMT) ratio of correlations (Henseler et al., 2015). The HTMT values across all construct pairs ranged from 0.20 to 0.53. Because all values were well below the conservative threshold of 0.85 (Henseler et al., 2015), discriminant validity is firmly established, confirming that the constructs measure distinct concepts.

Furthermore, to confirm the superiority of our hypothesized measurement model, we tested several alternative models (Table 3). The hypothesized four-factor model (benevolent climate, principled climate, ethical behavior, and innovative behavior) yielded the best fit to the data ( $\chi^{2}=172.34$ , df=128 , CFI = 0.96, TLI = 0.95, RMSEA = 0.04, SRMR = 0.05). We compared this against a three-factor model, a two-factor model, and a single-factor model (where all items were loaded onto one latent factor). As shown in Table 3, the one-factor model exhibited poor fit ( $\chi^{2}=411.00$ , df=135 , CFI = 0.71, RMSEA = 0.10). The fit indices progressively worsened as constructs were merged, confirming that our four-factor model is the most appropriate and providing further evidence against severe common method variance (Podsakoff et al., 2003).

**Table 3:** Comparison of measurement models.

Models	$\chi^{2}$	*df*	$\chi^{2}/\mathrm{df}$	CFI	TLI	RMSEA	SRMR
Hypothesized 4-factor model	172.34	128	1.35	0.96	0.95	0.04	0.05
3-Factor model	226.46	132	1.72	0.90	0.88	0.06	0.06
2-Factor model	367.63	151	2.43	0.78	0.75	0.08	0.09
1-Factor model	411.00	135	3.04	0.71	0.67	0.10	0.10

## 4 Data analysis and results

Since all the data was collected from the same source, there might be common method bias. So, we conduct multicollinearity and Harman’s single-factor tests to see whether method bias exists. We checked “tolerance” and “VIF” (variance inflation factor) values for each predictor as a check for multicollinearity. In our case, all the variables have greater than 0.10 tolerance values, and all VIF values were below 2. According to our results, multicollinearity among independent variables is unlikely (Hair et al., 2010). Harman’s single-factor test also shows that the consequence explains only 28% of the variance, confirming multicollinearity test results.

### 4.1 Correlation analysis

The descriptive statistics and correlations for the variables are presented in Table 4. Age and work experience were not significantly correlated with innovative behavior (*β = 0*.102 and *β = 0*.063, respectively). However, gender was negatively correlated with innovative behavior (*β = −0*.142, *p < 0*.05). Benevolent climate and principled climate were both significantly correlated with both ethical behavior (*β = 0*.162, *p < 0*.05 and *β = 0*.324, *p < 0*.01, respectively) and innovative behavior (*β = 0*.379, *p < 0*.01 and *β = 0*.362, *p < 0*.01, respectively). Ethical behavior was also significantly correlated with innovative behavior (*β = 0*.208, *p < 0.*01).

**Table 4:** Correlations and descriptive statistics.

Variables	Mean	SD	1	2	3	4	5	6
Age	2.034	1.009	1					
Gender	0.272	0.446	−0.151^*^	1				
Work experience	2.592	0.996	0.567^**^	−0.122	1			
Benevolent climate	3.939	0.708	−0.028	−0.137	−0.078	1		
Principled climate	4.027	0.662	0.053	−0.046	−0.044	0.426^**^	1	
Ethical behavior	3.646	0.694	0.076	−0.036	−0.025	0.162^*^	0.324^**^	1
Innovative behavior	3.992	0.678	0.102	−0.142^*^	0.063	0.379^**^	0.362^**^	0.208^**^

*n* = 206. Gender: 0 = male, 1 = female. **Correlation is significant at the 0.01 level (2-tailed), *Correlation is significant at the 0.05 level (2-tailed).

### 4.2 Regression analysis

To test our proposed hypotheses, we run regression analysis using IBM SPSS 23 (see Table 5 for the results). Hypothesis 1a predicted that a benevolent climate is positively related to ethical behavior. Hypothesis 1b predicted that principled climate is positively associated with ethical behavior. As shown in Table 5, Model 2, benevolent climate positively relates to moral behavior. Still, not significantly (*β* = 0.029, *t* = 0.39). However, principled climate is positively and significantly related to ethical behavior (*β* = 0.303, *t* = 4.099, *p* < 0.001). Therefore, while there was no support for H1a, there was support for H1b.

**Table 5:** Regression analysis results.

Variable	Model 1		Model 2		Model 3		Model 4	
	Innovative behavior		Ethical behavior		Innovative behavior		Innovative behavior	
	*β*	*T*	*β*	*t*	*β*	*t*	*β*	*t*
Age	0.054	0.699	0.096	1.171	0.056	0.674	0.045	0.586
Gender	−0.079	−1.222	−0.011	−0.164	−0.123	−1.788	−0.078	−1.209
Work experience	0.055	0.714	−0.065	−0.804	0.021	0.256	0.061	0.792
Benevolent climate	0.270^***^	3.843	0.029	0.39			0.268^***^	3.812
Principled climate	0.242^***^	3.467	0.303^***^	4.099			0.215^**^	2.957
Ethical behavior					0.200^**^	2.92	0.091	1.364
*R^2^*	0.210		0.112		0.066		0.217	
Adjusted *R^2^*	0.190		0.090		0.048		0.194	
*F*	10.633^***^		5.063^***^		3.571^**^		9.209^***^	

*n* = 206. Gender: female = 1, male = 0. ^*^p < 0.05. ^**^p < 0.01. ^***^p < 0.001.

Hypothesis 2 predicted that ethical behavior is positively related to innovative behavior. We conducted regression analysis to test this relationship. As shown in Table 5, Model 3, ethical behavior is positively related to innovative behavior and the relationship is significant (*β* = 0.200, *t* = 2.920, *p* < 0.01). Therefore, H2 was strongly supported.

### 4.3 Structural equation modeling (SEM) and mediation analysis

To test the mediation hypotheses (H3a and H3b) and address modern methodological standards, we conducted Structural Equation Modeling (SEM). SEM results indicated that benevolent climate did not significantly influence ethical behavior ( β=0.030,p=0.685 ), whereas principled climate significantly and positively influenced ethical behavior ( β=0.312,p<0.001 ). Crucially, when accounting for the climate dimensions in the full structural model, ethical behavior did not significantly predict innovative behavior ( β=0.072,p=0.319 ). Consequently, we tested the indirect effects using bootstrapping. The indirect effect of benevolent climate on innovative behavior through ethical behavior was not significant (Indirect effect = 0.002, p=0.707 , 95% CI [−0.009, 0.013]). Likewise, the indirect effect of principled climate was not significant (Indirect effect = 0.022, p=0.332 , 95% CI [−0.023, 0.068]). Because both 95% confidence intervals included zero, the mediation effects were not supported. Therefore, H3a and H3b are rejected.

## 5 Discussion

This study investigated the relationship between ethical climate types (benevolent and principled) and innovative behavior of public sector employees from the perspective of their perceived ethical behavior. We surveyed the public sector to find evidence of the relationship between ethical climate and innovative behavior. The most important part of this research is to conduct a study in Uzbekistan, where research in this field is lacking.

This study has derived several critical findings that refine our understanding of organizational climates. First and foremost, contrary to our initial mediation hypotheses, we found that ethical behavior does not mediate the relationship between ethical climate and innovative behavior. Instead, our results revealed that a principled ethical climate acts as a robust direct driver for both ethical behavior and innovative behavior independently. While basic regressions suggested a positive link between ethical behavior and innovation, this relationship became non-significant in the full structural model. This is a significant theoretical revelation: it suggests that adherence to ethical rules and engagement in innovative risk-taking are parallel, independent outcomes of a highly principled environment, rather than a sequential chain of behaviors. No study thus far has empirically unpacked this parallel mechanism in the literature, providing a vital starting point for future research.

Moreover, we confirmed the results of previous studies that found a link between ethical climate and ethical behavior (Deshpande, 1996; Deshpande and Joseph, 2009; Peterson, 2002; Fritzsche, 2000). Our results show that principled climate has a significant positive impact on ethical behavior. It can be inferred that organizations that run rules and procedures effectively may expect employees to act ethically. In other words, if employees perceive their organization’s rules and standard operating procedures better, they are more likely to act in line with ethical behavior. On the other hand, a benevolent climate was not found to have a significant relationship with moral behavior. As we know, in a benevolent climate, every member of the organization is concerned with the well-being of other members.

Another important finding is that our respondents’ mean value (*X̅* = 3.992) showed high results for innovative behavior. It is the opposite of the literature stream “stereotype” on public employees being “less innovative” (Bysted and Hansen, 2015; p. 698) under some circumstances. Even though we do not obtain the information about private sector employees of Uzbekistan, we believe that at least this result indicates they are not “less innovative.”

Regarding the structural relationships, this research definitively showed that ethical behavior does not mediate the link between ethical climates and innovative behavior. Rather than ethical behavior acting as a stepping stone to innovation, we found that a principled climate directly yields both outcomes simultaneously. For managers, particularly in public organizations where principled climates are dominant, this implies that establishing clear, rule-based ethical guidelines does not stifle creativity; rather, it creates a structured environment where employees feel secure enough to innovate while simultaneously remaining ethical. The lack of mediation also tells managers that they cannot simply rely on hiring ‘ethical people’ to naturally foster innovation. Instead, they must cultivate a principled organizational climate, which serves as the foundational root for both ethical conduct and creative output.

Our final model (Model 4) demonstrates an $R^{2}$ of 0.217, indicating that our variables collectively explain 21.7% of the variance in innovative behavior among public sector employees. While this explanatory power is modest, it is highly meaningful in the context of organizational psychology, demonstrating that ethical parameters are tangible drivers of innovation.

### 5.1 Implications, limitations, and future research directions

Our results have practical implications for organizations and managers. These results emphasize creating a favorable climate for employees to behave ethically and, by doing so, to benefit the organizations they are working for. An organization is ethical if the people inside it are ethical. Moreover, organizations can encourage people to act ethically by incentivizing ethical behavior and disincentivizing those who behave unethically (James, 2000). Managers monitor the behavior of employees by the organization’s expectations of appropriate behavior, and they must respond quickly to minimize the impact of suspected ethical violations. Therefore, managers should realize their crucial role in shaping the ethical climate and affecting the ethical behavior of employees.

This study has several limitations. The first limitation of this study is the characteristics of the data collection. The data were collected by primary data using self-report questionnaires, which may increase the possibility of response set tendencies and standard method variance. We must note that we have applied some procedural precautions to reduce common method bias, such as guaranteeing confidentiality and protecting anonymity.

Second, while we utilized Harman’s single-factor test and procedural remedies (e.g., ensuring anonymity) to check for Common Method Variance (CMV), we acknowledge that Harman’s test has modern statistical limitations (Podsakoff et al., 2003). Future studies should utilize temporal separation or unmeasured latent method constructs (ULMC) to more rigorously control for CMV.

In addition to the common method biases, our study may have some limitations related, for instance, to its scope (only the employees working in the public sector) and content (only two types of ethical climate: benevolent-local and principled-local). Additionally, the diversity of organizations involved in this study could be another limitation. As mentioned earlier in the data collection part, we collected data from various public sector organizations, and the employees’ innovative behavior in these organizations may differ. For instance, the innovative behavior of the employees working for research centers and institutes may vary from that of those working for ministries and joint-stock commercial banks. Also, the results cannot be strictly construed to represent all employees in the public sector worldwide, since this study has been conducted only in Uzbekistan. Therefore, the analysis must be conducted in private organizations and countries (particularly developing countries) to generalize the findings. Since the organizations in which this study was done are heavily male-dominated (72.3%), the results may differ in organizations with a larger proportion of females.

Considering the limitations, new and more developed research settings may be designed. As stated, this research aimed to investigate the relationship between ethical climate types and innovative behavior, with the mediating role of ethical behavior in the public sector. For the upcoming studies, examining the effect of other ethical climates on innovative behavior is possible. Also, in further research, the unit of analysis may be organizations rather than individuals, and the sample may be enlarged to the private sector. Another suggestion for future research could be to conduct a comparative study of the effect of the ethical climates of the public and private sectors on innovative behavior. It is also possible to extend the theoretical model by examining other variables: (1) dependent variables such as codes of ethics and ethical culture instead of ethical climate, and (2) ethical behavior as a moderator. Future research may also examine the effects of ethical climate types on the phases of innovation, such as idea generation, idea promotion, and idea implementation.

It is also recommended that more research on ethics and innovative behavior should be conducted in developing countries, “because it cannot be taken for granted that the same patterns of behavior and attitudes that exist in developed nations also exist in developing countries” (Okpara and Wynn, 2008; p. 947). The lack of research in this stream of literature in these countries creates a vacuum in the knowledge base of top management and corporate leaders. Furthermore, conducting such studies in these countries can contribute to these nations’ economic development and growth.

To conclude, it is always a challenge for people to start doing something new. Researchers are not the exception, and it is more challenging to take the first steps toward a new topic or area than to confirm already existing results. Although we predicted these challenges, we succeeded in filling the gap in this stream of research in Uzbekistan. Despite the above-mentioned limitations, this research has some important conclusions for researchers and organizations in Uzbek organizations and opens up a new avenue of research in ethics. The current study provides important preliminary insights on the orientation of ethical climates and their relationship with innovative behavior of public sector employees in Uzbekistan. This study is the first quantitative study that investigates the relationship between ethical climate and innovative behavior via the moral behavior of public sector organizations. We hope that the findings and implications of this research will provide productive ground to raise advanced and directed research.

## Data Availability

The raw data supporting the conclusions of this article will be made available by the authors, without undue reservation.
